# New models to investigate complex glucocorticoid receptor functions

**DOI:** 10.3389/fnbeh.2012.00090

**Published:** 2012-12-31

**Authors:** Christoph Anacker, Carmine M. Pariante

**Affiliations:** Department of Psychological Medicine, Section of Perinatal Psychiatry and Stress, Psychiatry and Immunology (SPI-Lab), Institute of Psychiatry, King's College LondonLondon, UK

**A commentary on**

**A zebrafish model of glucocorticoid resistance shows serotonergic modulation of the stress response**

by Griffiths, B. B., Schoonheim, P. J., Ziv, L., Voelker, L., Baier, H., and Gahtan, E. (2012). Front. Behav. Neurosci. 6:68. doi: 10.3389/fnbeh.2012.00068

The significance of the glucocorticoid receptor (GR) in stress response regulation and mood disorders has been demonstrated by many studies in animal and human models (Anacker et al., 2011a). The paper by Griffiths' and colleagues adds to this evidence, by investigating GR function and its role in stress response regulation and antidepressant action in an exciting new zebrafish model.

The main finding of this study is that mutant zebrafish larvae with impaired GR transactivation, due to a single nucleotide substitution in a region essential for DNA binding (gr^s357^ mutants), exhibit behavioral abnormalities and neuroendocrine dysfunction. Specifically, gr^s357^ mutants show elevated startle responses, a surrogate measure of anxiety-like behavior in fish, as well as increased cortisol levels and impaired HPA axis feedback control, demonstrated by reduced suppression of pituitary *pomc* upon treatment with the synthetic glucocorticoid, betamethasone. The authors also test whether the selective serotonin reuptake inhibitor (SSRI) antidepressant, fluoxetine, is able to reverse the neuroendocrine and behavioral phenotype of gr^s357^ mutants. Their findings suggest that fluoxetine does not reverse neuroendocrine abnormalities resulting from impaired GR transactivation, while it still effectively counteracts anxiety-like behavior. These findings confirm the crucial role of the GR in neuroendocrine regulation and behavioral stress responses, and are in line with the authors' previous work, in which they have identified stress axis abnormalities and increased freezing behavior upon exposure to a novel environment in the same mutant fish (Ziv et al., 2012). Their new findings thus consolidate the use of the zebrafish model to investigate GR-dependent regulation of the stress response.

The data suggest that some of the behavioral effects of fluoxetine on reversing startle response abnormalities may be independent of GR transactivation in fish. However, it is important to note that many GR-mediated effects are indeed *independent of direct GR binding to the DNA*, particularly the glucocorticoid-mediated regulation of *pomc* expression (Tuckermann et al., 1999; Bilodeau et al., 2006). It thus remains to be elucidated whether GR protein-protein interactions, such as transrepression of the transcription factors NFkB, AP-1, or cyclic AMP response element binding protein (CREB), may indeed account for some of the SSRI effects on anxiety-like behavior in this study. Moreover, an increasing body of work suggests that a membrane-bound GR may mediate some of the rapid, non-genomic effects of glucocorticoids, particularly glucocorticoid-induced changes in neuronal excitability and memory consolidation (for comprehensive reviews, please see: Haller et al., 2008 and Groeneweg et al., 2012). While previous studies have shown a crucial role for genomic GR effects in antidepressant action (Anacker et al., 2011b), the involvement of the membrane-bound form of the GR in the mechanism of action of antidepressants remains elusive and may possibly account for some of the behavioral changes upon antidepressant treatment in gr^s357^ mutant fish, in which only GR transactivation is abolished. As the authors suggest, the role of serotonergic signaling on the neuroendocrine system, behavior, and the regulation of GR function is likely to be of great importance for the effects of SSRI antidepressants (Laplante et al., 2002). At the same time, potential serotonin-independent molecular mechanisms of antidepressant action, such as liberation of G-proteins from membrane associated lipid rafts, have been suggested (Donati and Rasenick, 2005), and may represent possible alternative pathways involved in stress response regulation and GR activation, which warrant further investigation.

Of course, when modeling psychiatric disorders in fish, it needs to be carefully considered that the behavioral repertoire, the neural networks and the psychological responses to stress in teleosts are largely different from respective responses in the mammalian brain. Behavioral studies in higher animals, such as rodents and primates, as well as cellular and molecular studies in human tissues, will therefore indeed remain indispensable for our understanding of stress-induced brain abnormalities, depression pathophysiology, and antidepressant treatment response. To this end, it will be interesting for future investigations to examine if other well established neurobiological correlates of depression, such as alterations in neuronal gene expression or adult neurogenesis, can also be modeled in comparable regions of the fish brain (Kizil et al., 2012). In addition, evolutionary differences in corticosteroid receptor function have been demonstrated and need to be considered when investigating GR function across different phylogenetic taxa (Bury and Sturm, 2007). Nevertheless, the zebrafish is a useful model to investigate principle mechanisms underlying stress response regulation, and it offers a unique possibility for high-throughput drug screening *in vivo*, facilitating the discovery of new targets that alter neuroendocrine responses and proxy measures of stress-induced behavioral abnormalities.

In order to identify new treatment targets and to elucidate the sophisticated heterogeneous mechanisms underlying depression pathogenesis, a cross-species investigation into the several layers of the complex nature of stress-induced biological abnormalities will be essential to our success. The paper by Griffiths' and colleagues, together with previous work on GR function in zebrafish (Ziv et al., 2012), is a great example for such an approach, and offers a novel high-throughput platform in live fish to investigate drug responses and GR function in an *in vivo* system.
